# Additional Evidence Concerning Particular Drugs

**Published:** 1880-05

**Authors:** 


ADDITIONAL EVIDENCE CONCERNING PARTICULAR DRUGS.

*Argenti Nitras.*—Eight specialists consider it "unquestionably superior to any other astringent."

Dr. Green, of St. Louis, goes farther; he says, "Can do all with arg. nit. that I can do with *all* the others."

Dr. Webster, of New York, thus excellently summarizes a method of its use: "I more often use a ten-grain solution of arg. nit. than any other astringent. I apply it by a small mop of absorbent cotton twisted about the end of a dentist's cotton-holder or a slender piece of wood. In mild cases I apply the solution to a very small area of conjunctiva of upper lid, usually a little at the inner and a little at the outer end, where the redness is greatest. In severer cases I apply it to a still larger area, and in trachoma I apply it very thoroughly over the whole conjunctiva of upper and sometimes of lower lid. In all cases I neutralize with salt water before restoring lids to place."

*Advantages.*—1. Can produce with it any effect from the mildest astringent to the most powerful caustic. 2. Above tables show a greater percentage of success with it than with any other application. 3. It is painless except in strong solutions, or "mitigated stick," and even then can be rendered so by immediate neutralization with salt water.

*Disadvantages.*—1. It stains everything, even the conjunctiva sometimes, when used for a long time. 2. If injudiciously used it seems to do more harm than any other applications. 3. After long use scars of the conjunctiva are more apt to follow than after the use of other applications.

*Cupri Sulphas.*—Four specialists consider it "unquestionably superior to any other astringent."

Used in solution, but oftenest in solid crystal.

Very smooth and lightly applied to everted lids, as seen by table, ranks next to arg. nit. among specialists, and even among the advocates of the latter is sometimes chosen where conjunctiva is thin, and special liability to scars seems to exist. It is very convenient, and always ready for use. Its application is quite painful.

*Alumen.*—Used in crystal and in solution, ʒi. to water Oi.—suitable only for mild cases; crystal causes sharp pain, but for a few moments only.

*Zinci Sulphas.*—Used only in solution, grs. i.–v. to water ℥i, A great favorite as a collyrium, both among general practitioners and also with specialists as far as they use collyria.

*Hydrargyri Oxidum Flavum* (grs. ii. to vaseline ʒi.) is preferred by Dr. Seely, of Cincinnati, in trachoma, and in "all cases of chronic conjunctivitis," by Dr. Murdoch, of Baltimore.

*Formula*—℞ Zinci Sulphatis.
Cupri "
Ferri "
Aluminis, āā ʒi.

Dr. Holmes, of Chicago, prefers a saturated solution of the above for severe cases, and various solutions for the milder cases. Has used it largely and with great success. Solution is unstable.

*Tannin.*—Dr. W. S. Little, Philadelphia: "For chronic conjunctivitis without trachoma, tannin; for trachoma, tannin and copper. Along with tannin I generally insufflate calomel. Tannin and calomel are the principal articles which I use at the clinic and in private practice."

*Plumbi Subacetas.*—Dr. F. Buller, Montreal, Canada: "On the whole I think Plumb. Subacet. acts better than any other astringent.

The solution I employ is,

Liq. Plumb. Subacet., ℥i.

Aquae dest., ʒiii.

always applied by the surgeon himself to everted lids, and washed off with water."

Several correspondents say that they should use lead more were it not for its dangers; but the only danger mentioned is its liability to form deposits in corneal ulcers.

In addition to these eight favorite methods of treatment, fourteen other methods were mentioned with more or less favor.

Truly, here is not lack of material, and to sift that already on hand would be as profitable a task, perhaps, as to add to it.

In looking over the above evidence one conclusion is inevitably forced upon a candid mind, viz.; that there is no specific for chronic conjunctivitis.

Of course every observant man must by long practice acquire increased facility in the use of local applications, and must of necessity receive many impressions as to special indications, but no one as yet has placed them in such a form that any considerable number of specialists accept and act upon them.

Quite often, then, in commencing the use of local applications, but two courses are open:

1. Pure empiricism.

2. To have some favorite which on the whole seems to meet the largest number of cases.

Finally. The principal conclusion of the above inquiry is, that argenti nitras will meet a larger proportion of cases than any other local application.*

I return my sincere thanks to the gentlemen who have kindly furnished the basis of the above report. Many of the answers received are the summing up of twenty years' experience and over in special practice, and as such deserve publication in full. The necessity of brevity, however, will not permit.—*Proceedings of Connecticut State Medical Society, 1879.*

* All the local applications mentioned are not astringents; all are irritants, and some have caustic properties, but their practical value has been sought rather than their classification. The removal of any active cause for the conjunctivitis, *e. g.*, poor general condition, errors of refraction, strictures of lachrymal passages, etc., must of course precede any local application from which success is expected.

